# Pulse Pressure Relationships with Demographics and Kidney Function in Ashanti, Ghana

**DOI:** 10.1155/2018/7864564

**Published:** 2018-10-04

**Authors:** Debasish Banerjee, Jacob Plange-Rhule, Nihil Chitalia, Kwabena Kumi, Frank B. Micah, Francesco P. Cappuccio, John B. Eastwood

**Affiliations:** ^1^Department of Renal Medicine and Transplantation, St Georges, University of London, Cranmer Terrace, London SW17 0RE, UK; ^2^Department of Medicine, Komfo Anokye Teaching Hospital, P. O. Box 134, Kumasi, Ghana; ^3^Department of Renal Medicine, Darent Valley Hospital, Dartford, Kent DA2 8DA, UK; ^4^Division of Mental Health & Wellbeing, University of Warwick, Warwick Medical School, Gibbet Hill Road, Coventry CV4 7AL, UK

## Abstract

**Introduction:**

Hypertension, particularly pulse pressure [PP] is a major risk factor for end-stage renal disease. However, the effect of individual components of hypertension namely PP, systolic [SBP] and diastolic blood pressure [DBP] on kidney function, in the general African population is unknown.

**Methods:**

Data were collected on 944 participants [aged 40-75 y], living in villages in the area around the city of Kumasi, Ghana, on demographics, medications, height, weight, BP and 24-hour creatinine clearance (CrCl).

**Results:**

The demographic and clinical characteristics were: age 55(11) [mean (SD)] years, females 62%, rural village-dwellers 52%, diabetes 1·5%, BMI 21(4) kg/m^2^, 24-hourCrCl as a measure of glomerular filtration rate (GFR) 84(23) ml/min/1.73 m^2^. 29% had BP >140/90 mmHg; SBP and DBP were 125/74(26/14) mmHg, PP was 51(17) mmHg. PP increased with age by 0.55(95% CI: 0.46,0.64) mmHg/year. PP was higher (53(17) v 49(15) mmHg; p < 0.001) in the semiurban participants. GFR decreased both with increasing PP [-0.19 (-0.27,-0.10 ml/min/1.73 m^2^/mmHg; p < 0.001] and SBP [-0.09 (-0.14,-0.03) ml/min/1.73 m^2^/mmHg; p < 0.001] but there was no significant relationship with DBP [-0.04 (-0.15,0.06)]. After adjusting for SBP, the relationship between GFR and PP became steeper [-0.31 (-0.50,-0.12) ml/min/1.73 m^2^/mmHg; p < 0.001]. Using multivariate regression analysis that included PP, age, gender, BMI, only increasing age [-0.75 (-0.88,-0.62)] and decreasing BMI [0.49 (0.16,0.81)] were associated with decreased kidney function.

**Conclusions:**

In this homogeneous West-African population, PP increased with age and had a steeper relationship with declining kidney function than SBP or DBP.

## 1. Introduction

Hypertension is a major risk factor for chronic kidney damage, and while both systolic (SBP) and diastolic blood pressure (DBP) have been implicated as causal factors, it is now clear that SBP is a much stronger predictor of chronic kidney disease than DBP [1–3]. Isolated systolic hypertension (ISH) is becoming particular common especially in elderly, has also been recognised as a risk factor for cardiovascular disease [4–6]. In recent years there has been a growing interest in pulse pressure (PP) [systolic – diastolic pressure] as a marker of target end-organ damage [7]. Elevated PP is mainly due to central arterial stiffness and increases with age. The nature of PP and its association with demographics and kidney function in an African population has not been described.

PP has a stronger relationship than SBP alone with cardiovascular deaths in end-stage renal disease (ESRD) patients [3, 8, 9]. Also, in a predominantly Caucasian population with early and predialysis chronic kidney disease (CKD), we have found a strong relationship between PP and progression of chronic kidney disease, onset of dialysis, and death [2, 10].

Hypertension is common in Ghana, with estimated prevalence of about 29%, and hence a major cause of morbidity and mortality [11, 12]. However the effect of BP, particularly of PP, on kidney disease, in this population, is unknown. The present study examines the relationships between SBP, DBP, PP, demographic variables, and glomerular filtration rate (GFR), in a community study in villages in Ghana.

## 2. Methods

### 2.1. Participants

**Figure 1:**
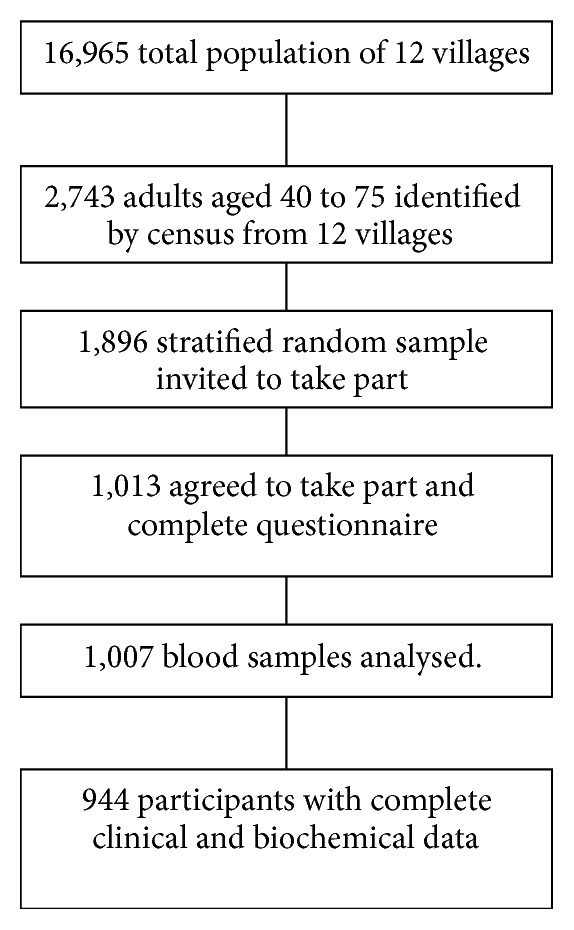
Recruitment of participants.

The present report uses data on 944 individuals [355 men, 589 women] from the 1,103 participants of the baseline survey for a cluster-randomised trial in 12 communities in the Ejisu-Juabeng and Kumasi Districts of the Ashanti Region, clustered around Kumasi, the second largest city in Ghana [11–13]. In this study “rural” refers to villages lacking any real infrastructure; they had no main water supply or sewage and often lacked electricity. These villages were up to 40 km from Kumasi. In contrast, most of the “semiurban” villages had main water and all had electricity; they were within 15 km of Kumasi. From each village, participants aged 40 to 75 were selected using age- and gender-stratified random sampling to ensure that participants from each village matched overall population structure [14]. Individuals with serious mental or physical illness, women pregnant or lactating, and individuals declining to participate were excluded from the study. Individuals with hypertension, diabetes or known renal disease were not excluded (Figure 1).

### 2.2. Questionnaire

Participants attended a registration centre in their village. After details of the study had been explained in the local language (Twi), individual written consent was obtained. All participants, with the help of a project assistant, completed the questionnaire, which included sections on demographic background, socioeconomic status, health, diet, lifestyle and past and current drug treatment. Ethical approval was granted by the Committee on Human Research Publication and Ethics of the School of Medical Sciences, Kwame Nkrumah University of Science and Technology, Kumasi, and by Wandsworth Research Ethics Committee, London.

### 2.3. Measurements

Methods are described in detail elsewhere [11–14]. In brief, height was measured to the nearest 0.5 cm using a wooden platform with height rule; footwear was removed. Weight was measured to the nearest 0.5 kg with manual Seca 761 scales (Vogel &Halke, Hamburg, Germany) after participants had removed outer garments and footwear. Blood pressure and pulse rate were measured with an automated sphygmomanometer (OMRON HEM705CP, Omron Matsusaka Co, Matsusaka City, Mie-Ken, Japan), using appropriate cuff size after participants had sat undisturbed for at least 5 minutes. Three readings were taken 1 minute apart. The first was discarded, and the mean of the last two used in the analysis. Hypertension was defined as a systolic BP ≥ 140 mm Hg and/or a diastolic BP ≥ 90 mm Hg or being on drug therapy for hypertension.

### 2.4. Blood Samples and 24-Hour Urine Collections

Venous blood samples were taken fasting from each participant. The samples were put into Vacutainer tubes and transported without delay to Komfo Anokye Teaching Hospital. The tubes were centrifuged at 1,500 rpm for 10 minutes at room temperature, and the serum transferred into 5 ml aliquot tubes, which were kept frozen at −20°C until analysed. Serum and urine creatinine were measured using a Jaffe alkaline picrate method (Beckman–Coulter). Our method has been validated against isotope dilution mass spectrometry (IDMS) values for creatinine measurement [11].

Two consecutive timed 24-hour urine collections were obtained from each participant and the excretion of sodium, potassium and creatinine clearance were measured.

### 2.5. Statistical Analysis

GFR was measured as the mean of two creatinine clearances based on 24-hour urine collections and adjusted for body surface area. 24-hour urinary sodium and potassium were also measured. Body mass index (BMI) was calculated from the equation: BMI = weight/height^2^ [kg/m^2^]. Data were analysed using SPSS v.17 (Chicago, SPSS Inc). Results are presented as mean ± SD or median (interquartile range) for continuous variables and relationships between variables using correlation and regression coefficients with 95% confidence intervals. A two-sided p value < 0.05 is taken as statistically significant.

## 3. Results

### 3.1. Participants (Table 1)

The mean age of the population was 54.6 (11.1) years. 62% were females and 49% rural village dwellers. Only 1.6% of the study population were self-reported diabetics. BMI was 21.1 (4.2) kg/m^2^, haemoglobin 12.2 (1.7) g/dl, and creatinine clearance 84 (22) ml/min/1.73 m^2^. 24-urine sodium/potassium ratio was 2.3 (1.2) mmol/mmol. The mean BP of the study population was 125/74 (26/14) mmHg with a PP of 51 (17) mmHg. The overall prevalence of hypertension in the population was 28.7%, which higher than in other parts of Africa [12], with 30% of men and 28% of women diagnosed with hypertension in this study. The prevalence of hypertension was high despite half of the studied population were rural dwellers with a lean body mass. However, only about 8% of men and 4% of women were on any pharmacological treatment for hypertension. PP was higher in the semiurban population than in the rural village dwellers (Table 1). The rural population also had a lower BMI compared to the semiurban population.

### 3.2. Blood Pressure, Age, and Gender

**Figure 2:**
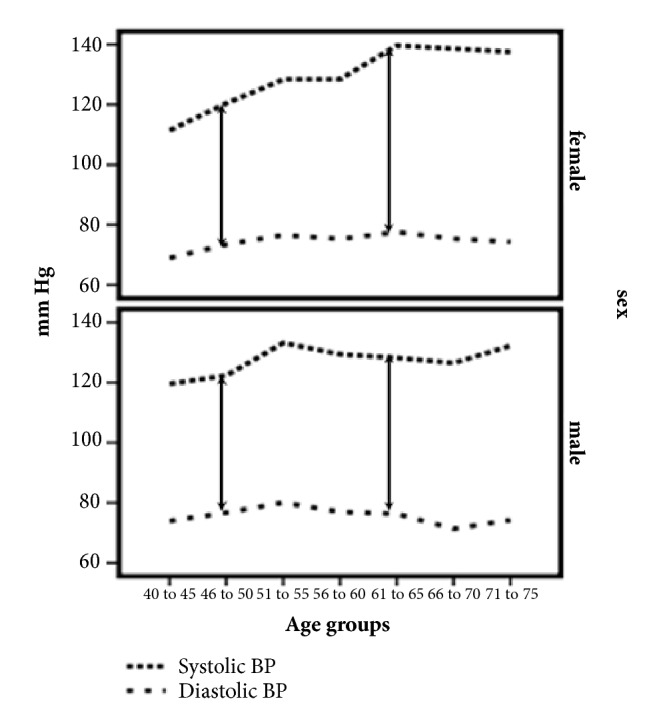
The effect of age on systolic and diastolic blood pressure in both genders. The top panel shows the changes in systolic (small dots) and diastolic (large dots) blood pressure variation with different age groups in females; the bottom panel shows the systolic and diastolic blood pressure variation with age in males.

**Figure 3:**
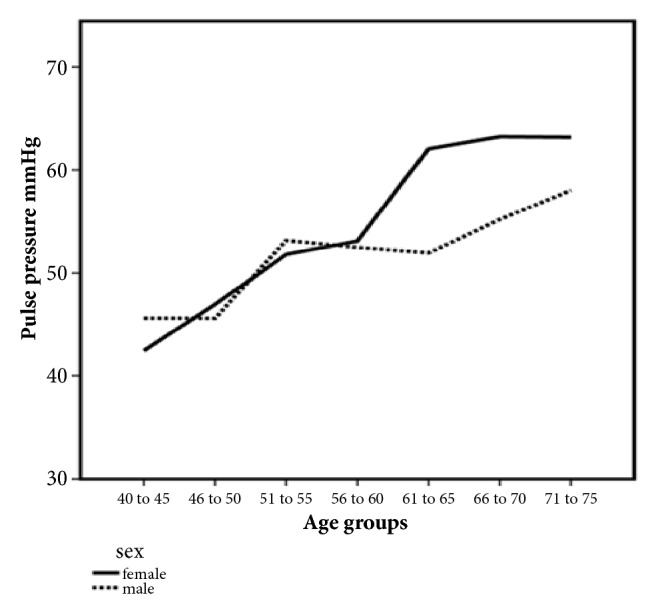
Changes of pulse pressure with age in males and females. The bold line shows the change in pulse pressure with different age groups in females; the dotted line shows the change pulse pressure with age in males.

SBP increased with age by 0.70 [95% CI (0.51 to 0.80); p < 0.001] mmHg/year in the overall population. PP also increased with age [0.55 (0.46 to 0.64, p < 0.001) mmHg/year]. DBP did not increase significantly with age particularly in subjects 55 years or older [-0.11 to -0.28 to 0.07) mmHg/year; p = 0.236]. The increase in blood pressure was seen in both men and women. However, a steeper rise of SBP and PP was noted in women than men at an older age (Figures 2 and 3 and Table 2). The women had a higher mean BMI than men.

### 3.3. Blood Pressure, Age, Gender, and Kidney Function

**Figure 4:**
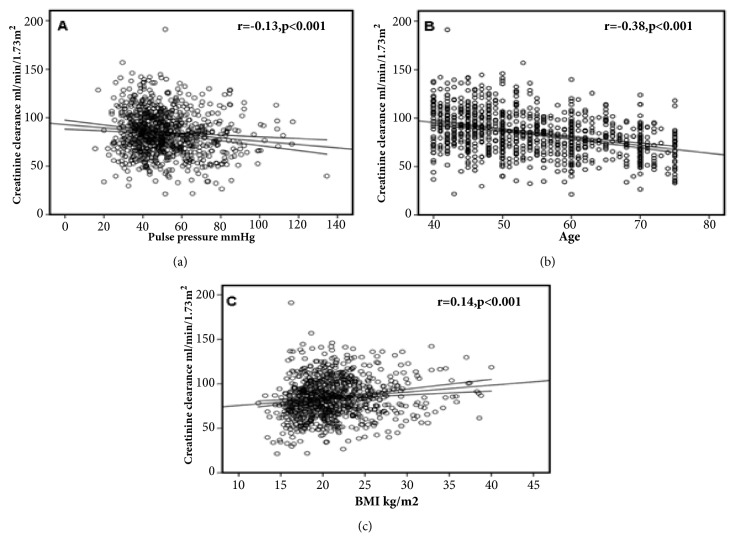
Associations of kidney function with pulse pressure, age, and body mass index. (a) demonstrates relationship between pulse pressure and creatinine clearance, (b) demonstrates relationship between age and creatinine clearance, and (c) demonstrates relationship between body mass index and creatinine clearance. The correlation coefficient and p values are shown with individual panels.

Creatinine clearance showed a strong inverse correlation with age; it also correlated inversely with PP and directly with BMI (Figure 4). On univariate analysis, creatinine clearance decreased with increasing PP [*β* = -0.18 (95% CI: -0.27 to -0.10) ml/min/1.73 m^2^/mmHg, p < 0.001] and SBP [*β* = -0.08(-0.14 to -0.03) ml/min/1.73 m^2^/mmHg, p = 0.002], but there was no significant association between creatinine clearance and DBP [*β* = -0.04 (-0.14 to 0.06) ml/min/1.73 m^2^/mmHg, p = 0.43] (Table 3). Creatinine clearance showed a slightly stronger inverse correlation with age in women (*β* = -0.399, p < 0.0001), compared to men (*β* = -0.29, p < 0.0001).

Using multivariate analysis, after adjusting for SBP the relationship between creatinine clearance and PP became stronger [-0.25 (-0.53 to -0.14) ml/min/1.73 m^2^/mmHg, p = 0.001]. In contrast, although creatinine clearance increased with an increase in SBP, the relationship no longer held after adjusting for PP [0.14 (-0.004 to 0.24) ml/min/1.73 m^2^/mmHg, p = 0.06].

There was no significant difference in creatinine clearance between men and women. [83 ± 21 v 85 ± 23 ml/min/1.73 m^2^, p = 0.16]. On multivariate regression analysis with creatinine clearance as dependent variable, and age, sex, pulse pressure, BMI and presence of diabetes as independent variables, only age [*β* = -0.75 (-0.88 to -0.62), p < 0.001] and BMI [*β* = 0.49 (0.16 to 0.81), p = 0.003] were independent predictors of creatinine clearance.

## 4. Discussion

This is the first study investigating the effects of age, gender, and demographic factors on blood pressure components, along with the effect of blood pressure on kidney function in a rural African population.

Our study shows that hypertension is common in Ghana. We have shown an increase in SBP and PP with age, in both men and women. Both SBP and PP rose with age, the rise being more marked in the women that in the men. Both SBP and PP were higher in the semiurban than in the rural population.

The increase in PP with age is well known, and has been ascribed to increasing central arterial stiffness. The trajectory of rise in PP with age may be gender related and is steeper in women than in men [15]. We have also seen a steeper rise of PP with age in women compared to men in our population, and believe that the higher BMI in women may be a possible explanation.

There is evidence that the higher PP in semiurban populations may be as a consequence of altered dietary habits and sedentary lifestyle leading to an increase in body weight [16]. In our population the higher PP and SBP in the semiurban population may also be explained by the higher BMI in these individuals. Interestingly, this trend of increasing blood pressure with urbanisation was not seen in a study from Tanzania [17].

PP is a better predictor of CVD than SBP and DBP among middle age Caucasian populations [18, 19]. In our previous studies, in a predominantly Caucasian population, PP was shown to be a good predictor of early chronic kidney disease (CKD) progression, and onset of dialysis, and death in predialysis CKD patients [2, 10]. The study aimed at identifying if pulse pressure is a good marker at predicting CKD, measured with 24 hour urinary creatinine clearance, in West-African rural and semiurban population. Unfortunately none of the accepted gold standard glomerular filtration rate (GFR) measurements were available to us during screening, and as demonstrated in our previous report, none of the current estimated GFR equations accurately predicted the GFR in this population based on 24-hour urinary creatinine clearance adjusted for body surface area. Hence this study measured renal function by the mean of two 24-hour creatinine clearance values.

In summary our study was directed to assessing the role of PP in predicting GFR. PP preformed slightly better than SBP, and both were highly correlated with GFR, unlike DBP, which showed no significant relationship. However, we have found age to be a significant confounder while assessing the impact of blood pressure parameters on GFR. The relationship of pulse pressure with decreasing kidney function was stronger than that with systolic blood pressure. Diastolic blood pressure had no relationship with kidney function.

## 5. Conclusion

This is the first population study showing gender and demographic differences in blood pressure parameters in a homogeneous West-African population from central Ghana. Sub-Saharan Africans have a higher PP when compared to Caucasians of similar age. We have also demonstrated that PP, a measure of arterial compliance, has a better relationship with declining renal function than measurement of either systolic or diastolic blood pressure. This study does provide support for the notion that in the prevention of cardiovascular disease (including stroke and chronic kidney disease) blood pressure reduction is a vital component of public health policy in Sub-Saharan Africa.

## Figures and Tables

**Table 1:** Clinical characteristics of the population.

	** Females *n = 589***	**Males *n = 355***	**Total *n = 944***
Age	54.8 (11.5)	54.5 (10.7)	54.6 (11.1)
BMI baseline (kg/m^2^)	21.7 (4.7)	20.2 (3.1)^*∗∗*^	21.1 (4.1)
Diabetics (%)	1.5%	1.7%	1.6%
Rural dwellers (%)	46.3%	51.5%	48.3%
SBP mean baseline (mmHg)	125.2 (27.1)	125.9 (24.1)	125.4 (26.0)
DBP mean baseline (mmHg)	73.6 (13.5)	75.6 (13.5)^*∗*^	74.4 (13.4)
Pulse pressure baseline (mmHg)	51.6 (17.5)	50.2 (14.7)	51.1 (16.5)
Serum Haemoglobin (g/dl)	11.7 (1.4)	13.1 (1.9)^*∗∗*^	12.2 (1.7)
Serum creatinine (*μ*mol/L)	73 (13)	90 (18)^*∗∗*^	79 (17)
Mean 24 hour urinary volume (Litres/day)	1.2 (0.5)	1.1 (0.5)	1.1 (0.5)
Mean 24 hour urinary sodium excretion (mmol/day)	103 (45)	100 (45)	102 (45)
Mean 24 hour urinary potassium excretion (mmol/day)	48 (22)	51 (26)^*∗*^	49 (23)
Urinary Sodium:potassium ratio (mmol/mmol)	2.4 (1.2)	2.2 (1.2)	2.3 (1.2)
24 hour mean creatinine clearance (ml/min/1.73 m^2^)	85 (23)	82 (22)	84 (23)

Mean and (SD) are given for all normally distributed variables.

†: Median and (IQR) for skewed variables (NS).

^*∗*^for p < 0.05.

^*∗∗*^for p < 0.005.

**Table 2:** Pulse pressure and age relationships in females and males according to age.

	***β***	**95**%** Confidence interval**	**P value**
**Age < 55 years**			
Female	0.8	(0.4-1.0)	<0.001
Male	0.5	(0.1-0.8)	0.008

**Age > 55 years**			
Female	0.44	(0.1-0.7)	0.007
Male	0.36	(-0.5-0.7)	0.84

**Table 3:** Relationship of different blood pressures with kidney function (creatinine clearance).

	***β***	**95**%** Confidence interval**	**p value**
**Univariate analysis **			
** PP**	-0.18	(-0.27 to -0.10)	<0.001
** SBP**	-0.08	(-0.14 to -0.03)	0.002
** DBP**	-0.04	(-0.14 to 0.06)	0.43

**Bivariate analysis**			
** PP**	-0.31	(-0.50 to -0.12)	<0.001
** SBP**	0.09	(-0.03 to 0.21)	0.143
** PP**	-0.22	(-0.32 to -0.12)	<0.001
** DBP**	0.09	(-0.03 to 0.21)	0.143
** SBP**	-0.22	(-0.32 to -0.12)	<0.001
** DBP**	0.31	(0.12 to 0.50)	<0.001

## Data Availability

The data used to support the findings of this study are available from the corresponding author upon request.
